# The British Journals: Medical Statistics

**Published:** 1840-01

**Authors:** 


### STATISTICS.

### *Statistics of Poisoning in England.*

[We are indebted for the following important digest to a respectable provincial newspaper: it is taken from a parliamentary document, entitled, "Returns from the Coroners of England and Wales of all Inquisitions held by them during the years 1837 and 1838, in cases where Death was found, by Verdict of Jury, to have been caused by Poison." We regret, with our contemporary, first, that the Returns are not complete, some Coroners having neglected to comply with the request of the Commons; and, secondly, that the Coroners who have made Returns have, in many instances, omitted particulars of great moment. The following particulars are derived from the returns of 41 counties and 31 boroughs only. The total number of deaths, it will be seen, are 543, viz. 282 males, and 261 females.]

*Arsenic* . . . . 184

- Taken by a girl, disappointed in love 1
- By a girl, in a fit of passion . 1
- By a girl, in a fit of jealousy . 1
- By a girl, who had robbed her master's son . . . . 1
- By a girl, seduced and deserted by a married man . . . 1
- By a girl, subject to fits and despondency . . . 1
- By pregnant girls, to destroy themselves . . . . 5
- By a pregnant girl, to procure abortion . . . 1
- By a pregnant girl, deserted by her lover, who was suspected to have procured her the poison . 1
- By a pregnant girl—how or by whom administered not known . 1
- By a wife, separated from her husband . . . . 1
- By a young woman, married unhappily, and separated from her husband . . . 1
- By a cook maid, distressed by the death of a friend . . 1
- By an insane mother and two children—administered by the former . . . . 3
- By five children, to whom it was administered by an insane mother 5
- By a man, embarrassed by debt, and disordered in mind and body 1
- By men, through reduced circumstances, pecuniary embarrassments, &c. . . . 6
- Taken through drunkenness 12
- By a farmer and innkeeper, who, having had a handsome legacy left to him, spent it in riotous living—got into debt—and took poison to escape his creditors . 1
- Through poverty . . . 3
- Through despondency . . 1

*Arsenic.*

- In lunacy . . 52
- In food, by accident . . 7
- In mistake, by young people, in food prepared for vermin . 5
- In mistake, by a married woman, who, having mixed it with oatmeal for vermin, was innocently supplied by her husband with food prepared from the mixture . 1
- By accident, the deceased having tobacco and arsenic loose in the same pocket . . . 1
- In mistake for cream of sulphur . 1
- Administered to a child in mistake for magnesia . . . 1
- ———— Queen's cordial . . 1
- Taken inadvertently, in various ways 3
- Administered wilfully . . 5
- How administered not known . 2
- Felo de se . . 20
- Taken without cause assigned in the report . . 36

*Opium* . . . . . 42

- Overdose, taken by adults in ignorance . . . 11
- Overdose, administered to children by mothers and nurses . . 8
- Administered to a child in mistake for other medicine . . 1
- Supplied by a deaf druggist for manna, and administered to a child by an ignorant nurse . . 1
- Administered to a child, found dead in the Trent, extensively bruised, (the poison and the wounds both sufficient to account for death) . 1
- Taken by a child in ignorance . 1
- Taken through drunkenness . 2
- Through lunacy . . . 9
- How administered not known . 1
- Felo de se . . . 2
- Taken without cause assigned, &c. . . . . 5

*Laudanum* . . . . 133
Administered by mistake . . 2
——— for antimonial wine . 1
——— for paregoric . . 2
——— for Godfrey's cordial . 2
——— for syrup of buckthorn . 1
——— for tincture of rhubarb . 4
Sold at a druggist's for antimonial wine—the druggist not bred to his trade, and kept two shop-girls, one of whom (the coroner ascertained) gave twice as much laudanum for a penny as the other . 1
Taken by adults as medicine 11
An overdose, taken by a drunken surgeon . . . . . 1
Taken by mistake for a surgeon's draught . . . . 1
Administered to children in mistake 2
Drunk by a child, within whose reach the phial had been left . 1
Given by a child to an infant, to allay coughing, in the mother's absence . . . . . 1
Overdose to infants by mothers and nurses . . . . 26
Taken inadvertently . . 7
Through despondency . . 4
Through drunkenness . . 9
Through dissolute conduct . 1
Through lunacy, induced by want . 2
Through do., from various causes 30
Through loss of situation . . 1
Wilfully administered . . 2
How administered not known . 3
Felo de se . . . . 4
No cause assigned, &c. . 14

*Cough Syrup* . . . . 1
Overdose, given by a mother to her child . . . . . 1

*Syrup of Poppies* . . . 5
Overdose, administered to children by mothers and nurses . . 5

*Godfrey's Cordial* . . . 12
Overdose, administered to children by mothers and nurses 10
Administered to children by mistake for syrup of rhubarb . 2

*Infant's Mixture (most probably a preparation of Opium)* . . 1
Overdose, given by a mother to her child . . . . 1

*Morrison's Pills* . . . 1
Taken as medicine . . 1

*Tartar Emetic* . . . . 2
Three drachms, taken to cure the ague . . . . . 1
Overdose, given to an infant . 1

*Colchicum* . . . . . 3
Overdose, taken for the gout . 1
Taken as medicine . . 2

*Mixture for vermin* . . . 2
Taken by children, within whose reach it was left . . 2

*Hellebore* . . . . . 1

Taken by a "temporary lunatic" . 1

*Mercury* . . . . . 2

Taken by a "temporary lunatic" . 1

Felo de se . . . . 1

*Bichromate of Potash* . . . 1

Eaten ignorantly by a child . 1

*Aquafortis* . . . . . 2

Drunk by a child within whose reach it was left . . 1

Taken in temporary lunacy . 1

*Oxalic acid* . . . . . 19
Taken by a woman, who had quarrelled with her husband . 1
By a person of defective intellect . . . . 1
Through lunacy . . . 8
Through drunkenness . . 1
Through want of employment . 1
By a young woman, on the emigration of her brother . . 1
By a child within whose reach it was left . . . 1
Without cause assigned . . 5
*** It is singular, that nearly the whole of the cases of poisoning by oxalic acid occurred in Middlesex

*Nitrate of Silver* . . . 1
By a child (swallowed percussion caps) . . . . 1

*Castor-Oil Seeds* . . . 1

Taken inadvertently . . 1

*Fungus* . . . . . 4

Eaten for mushrooms . . 4

*Rum* . . . . . 1
Ignorantly given to a child for inflammation of the bowels . 1

*Extract of Lead* . . . 1
Found in solution by a woman, and given to her child in mistake for ginger-wine . . . 1

*Essential Oil of Almonds* . . 4
Taken in lunacy . . 2
Without cause assigned . . 2

*Prussic Acid and Arsenic* . . 1
Taken in lunacy . . . 1

*Arsenious Acid* . . . 1
Taken in mistake for a purging powder . . . . 1

*Acetate of Morphine* . . . 2
Administered in mistake for other medicine . . . 2

*Strychnine (the active principle of Nux Vomica)* . . . 2
Taken by a child, to whose father it had been sent as a medicine . 1
Lunacy . . . . 1

*Nux Vomica* . . . . 3
Taken in ignorance of its effects . 1
Procured by a girl of weak intellect, and given to her father, who had sent her for an emetic . . 1
Taken without cause assigned . 1

| | | |
|---|---|---|
| *Wolf's Bane* | | 1 |
| Eaten by a child, who found it in his father's garden | 1 | |
| *Black Ashes* | | 1 |
| Procured for washing, and eaten by a child | 1 | |
| *Sulphate of Iron (Copperas)* | | 1 |
| Taken to procure abortion | 1 | |
| *A Vegetable Poison* | | 3 |
| Taken by two children (brothers) | 2 | |
| By an adult | 1 | |
| *Hicrapicra* | | 1 |
| An overdose, taken in gin | 1 | |
| *Monk's Hood* | | 1 |
| Gathered by a poor old man, and eaten in mistake for celery | 1 | |
| *Savine* | | 1 |
| Taken to procure abortion | 1 | |
| *Infusion of Hemlock* | | 1 |
| Overdose, taken by a woman | 1 | |
| *Laudanum and Prussic Acid* | | 1 |
| A case of lunacy | 1 | |
| *Potash* | | 1 |
| Taken by a child | 1 | |
| *Medicine* | | 1 |
| Administered to an infant—intended for an adult | 1 | |
| *Muriate of Tin* | | 1 |
| Taken by a child in mistake for vinegar | 1 | |
| *Cantharides* | | 1 |
| An embrocation, containing tincture of cantharides, administered to a child in mistake | 1 | |
| *Laudanum and Aquafortis* | | 1 |
| Lunacy | 1 | |
| *Carburetted Hydrogen Gas* | | 2 |
| Inhaled during sleep, through an accidental escape of gas | 2 | |
| *Belladonna (Deadly Nightshade)* | | 2 |
| Taken by mistake | 1 | |
| Without cause assigned | 1 | |
| *Paregoric Elixir* | | 2 |
| Overdose, administered to children | 2 | |
| *Decoction (nature not exactly known)* | | 1 |
| Taken by a pregnant girl, with the supposed intention to procure abortion | 1 | |
| *Nitrous Acid, with Aloes* | | 1 |
| Taken without cause assigned | 1 | |
| *Cayenne Pepper, &c.* | | 1 |
| Cayenne pepper, essential oil of Cayenne, and bark, taken in alcohol, as a remedy for the ague | 1 | |
| *Turberth Mineral* | | 1 |
| Taken in mistake | 1 | |
| *Sulphuric Acid (Vitriol)* | | 32 |
| Swallowed by children, ignorantly | 9 | |
| ——— for ginger beer | 3 | |
| Administered to children, for Godfrey's cordial | 4 | |
| ——— a child for castor oil | 1 | |
| ——— for syrup of rhubarb | 1 | |
| ——— for some medicine not named | 1 | |
| Accidentally sold for Godfrey's cordial, and given as such to a child | 1 | |
| In a drunken fit | 1 | |
| Through insanity | 5 | |
| Through family quarrels | 1 | |
| By a woman, who thought herself forsaken by God | 1 | |
| Without cause assigned | 4 | |
| *Hydrocyanic (Prussic) Acid* | | 27 |
| Taken by surgeons, depressed in mind by reduced circumstances | 2 | |
| By a surgeon, delirious from scarlet fever | 1 | |
| By a surgeon, addicted to drinking | 1 | |
| By a surgeon, in a fit of frenzy | 1 | |
| By druggists, deranged | 2 | |
| By a medical student, affected by over-study | 1 | |
| By a child, in ignorance | 1 | |
| By a gentleman, reduced from affluence to poverty, and deranged | 1 | |
| Through disappointment in love | 1 | |
| Through lunacy | 9 | |
| Without cause assigned | 6 | |
| *Corrosive Sublimate* | | 12 |
| Taken incautiously as medicine | 1 | |
| By mistake, for cider | 1 | |
| In a fit of passion | 1 | |
| Through despondency | 1 | |
| Through lunacy | 5 | |
| Felo de se | 2 | |
| Without cause assigned | 1 | |
| *Poisons not Specified* | | 14 |
| Taken accidentally | 2 | |
| By a drunkard, in mistake | 1 | |
| Case of miscarriage, the mother having received some noxious drug | 1 | |
| Taken without cause assigned | 2 | |
| Through lunacy | 7 | |
| How administered not known | 1 | |
| | | 543 |

*The Gateshead (Newcastle) Observer, Nov.* 2, 1839.

